# Impact of post-processing methods on apparent diffusion coefficient values

**DOI:** 10.1007/s00330-016-4403-6

**Published:** 2016-06-01

**Authors:** Martin Georg Zeilinger, Michael Lell, Pascal Andreas Thomas Baltzer, Arnd Dörfler, Michael Uder, Matthias Dietzel

**Affiliations:** 1 Institute of Diagnostic Radiology, University of Erlangen-Nuremberg, Maximiliansplatz 1, D-91054 Erlangen, Germany; 2 Department of Radiology and Nuclear Medicine, Medical University Vienna, Währinger Gürtel 18-20, 1090 Vienna, Austria; 3 Department of Neuroradiology, University of Erlangen-Nuremberg, Schwabachanlage 6, D-91054 Erlangen, Germany

**Keywords:** DWI, ADC, Reproducibility, MRI, Tumour

## Abstract

**Objective:**

The apparent diffusion coefficient (ADC) is increasingly used as a quantitative biomarker in oncological imaging. ADC calculation is based on raw diffusion-weighted imaging (DWI) data, and multiple post-processing methods (PPMs) have been proposed for this purpose. We investigated whether PPM has an impact on final ADC values.

**Methods:**

Sixty-five lesions scanned with a standardized whole-body DWI-protocol at 3 T served as input data (EPI-DWI, b-values: 50, 400 and 800 s/mm^2^). Using exactly the same ROI coordinates, four different PPM (ADC_1–ADC_4) were executed to calculate corresponding ADC values, given as [10^-3^ mm^2^/s] of each lesion. Statistical analysis was performed to intra-individually compare ADC values stratified by PPM (Wilcoxon signed-rank tests: α = 1 %; descriptive statistics; relative difference/∆; coefficient of variation/CV).

**Results:**

Stratified by PPM, mean ADCs ranged from 1.136–1.206 *10^-3^ mm^2^/s (∆ = 7.0 %). Variances between PPM were pronounced in the upper range of ADC values (maximum: 2.540–2.763 10^-3^ mm^2^/s, ∆ = 8 %). Pairwise comparisons identified significant differences between all PPM (*P* ≤ 0.003; mean CV = 7.2 %) and reached 0.137 *10^-3^ mm^2^/s within the 25th–75th percentile.

**Conclusion:**

Altering the PPM had a significant impact on the ADC value. This should be considered if ADC values from different post-processing methods are compared in patient studies.

***Key Points*:**

• *Post-processing methods significantly influenced ADC values*.

• *The mean coefficient of ADC variation due to PPM was 7.2 %*.

• *To achieve reproducible ADC values, standardization of post-processing is recommended*.

**Electronic supplementary material:**

The online version of this article (doi:10.1007/s00330-016-4403-6) contains supplementary material, which is available to authorized users.

## Introduction

Diffusion-weighted imaging (DWI) has become an indispensable tool for the examination of the central nervous system, and is increasingly used in body radiology. In proton MR imaging, extracellular water diffusion primarily contributes to measurable diffusivity. Further, capillary perfusion and molecular motion due to other causes, such as pressure or thermal gradients, also influence measured diffusivity values. As a consequence, quantitative results of DWI measurements are referred to as an apparent diffusion coefficient (ADC) [1].

Typically, lower ADC values are observed in malignant tumours compared to healthy tissue [2, 3]. This is usually explained by microstructural differences, such as an increased cellularity in malignant tumours. Typical examples of false-positive cases are glandular structures in adenocarcinomas or colliquative necrosis [4, 5].

In clinical practice, the ADC is assessed using parametric maps. However, the generation of such maps is not straightforward. It requires post-processing of raw DWI data, and multiple post-processing methods (PPMs) have been published for this purpose. Notably, many ADC researchers have used software tools provided by the vendor. Such tools are frequently proprietary and thus details of the algorithms are not generally available to users [3, 6].

This calls into question the reproducibility of ADC values. Therefore, we aimed to investigate whether PPMs have an impact on the ADC value.

## Methods

### Patients

We chose 25 patients (mean age 58 years, range 37–81 years) randomly from our prospectively populated institutional PET-MRI database. The latter contains patients with various oncological diseases of advanced stages. Thus, histological verification, imaging follow-up and interdisciplinary tumour board consensus were defined as the standard of reference (SOR). Details on patient diagnosis are summarized in Table 1.

**Table 1:** Details on type and site of lesions

Disease	Patients	LN	No LN
Lung cancer	10	10	21
Neuroendocrine neoplasm	5		7
Gastrointestinal neoplasm	3		4
Lymphoma	2	8	
Breast cancer	1	6	
Cancer of unknown primary	2	4	
Ovarian carcinoma	1		1
Thyroid carcinoma	1	4	
*Total*	*25*	*32*	*33*

*Note* A total of 65 malignant lesions in 25 patients were included. Thirty-two lesions where located within lymph nodes (LN) of which eight were lymphomas and 24 lymph node metastases. The remaining 33 lesion consisted of 12 organ metastases (liver, brain, bone, etc.) and 21 primary tumours

Such inclusion criteria were used in order to create a patient collective that would cover the whole spectrum of ADC values, ranging from about 0.2 (lymph nodes, bone marrow) to 2.4 * 10^-3^ mm^2^/s (kidney cortex [2, 7]).^1^

### Imaging

All patients were examined on a 3-Tesla Biograph mMR unit using phased array body coils (Siemens Healthcare Division, Erlangen, Germany). Patients thus received a whole-body (WB) examination at the Department of Radiology, University Hospital Erlangen, including morphological T1- and T2-weighted sequences and the DWI sequence.

The latter used WB, free-breathing, multiple-signal-acquisition EPI sequences (echo planar imaging) with three different b-values (50, 400 and 800 s/mm^2^). This DWI protocol followed recommendations for “Whole-Body Diffusion-weighted MR Imaging in Cancer” published by Padhani and colleagues in [3]. Technical details of this protocol are summarized in Table 2.

**Table 2:** Imaging parameters of the DWI sequence

Parameter	Value
Slice thickness	5 mm
Field of view	284 mm × 379 mm
Matrix	108 × 192
Repetion time	9,200 ms
Effective echo time	82 ms
Fat saturation	SPAIR
Parallel imaging	GRAPPA: factor 2
Avarages	4
Slice orientation	axial

*GRAPPA* GeneRalized Autocalibrating Partial Parallel Acquisition, *SPAIR* Spectrally Adiabatic Inversion Recovery

### Post-processing methods (PPMs)

Four different PPMs were executed in every lesion, based on the same raw DWI data (i.e. the b50, b400 and b800 images). This approach allowed the creation of paired sets of ADC values to compare four PPMs on an intra-individual basis. The following PPMs were used:

- ADC_1: ADC map *generated automatically inline by the scanner* using b-values of 50, 400 and 800 s/mm^2^ (Biograph mMR, Siemens Healthcare Division, Erlangen, Germany)
- ADC_2: *Manual logarithmic calculation *[8]. ADC_2 was calculated using the signal intensity (SI) of raw DWI data at the given ROI position (see below): ADC_2 can thus be expressed as a function of SI at b50 and b800 as follows:
  1 $$ ADC\_2=\frac{ln\frac{S{I}_{b800}}{S{I}_{b50}}}{b_{50}-{b}_{800}} $$
- ADC_3: *Manual ordinary least squares regression analysis* [9]. ADC_3 is a function of SI at b50, b400 and b800 as follows:
  2 $$ ADC\_3 = \frac{\left({b}_{50} - \overline{b}\right)*\left( \ln S{I}_{\mathrm{b}50} - \overline{ \ln SI}\right)+\left({b}_{400} - \overline{b}\right)*\left( \ln S{I}_{\mathrm{b}400} - \overline{ \ln SI}\right)+\left({b}_{800} - \overline{b}\right)*\left( \ln S{I}_{\mathrm{b}800} - \overline{ \ln SI}\right)}{{\left({b}_{50} - \overline{b}\right)}^2 + {\left({b}_{400} - \overline{b}\right)}^2+{\left({b}_{800} - \overline{b}\right)}^2} $$
  Where $\overline{b}$ equals the arithmetic mean of all three b-values (416.67 s/mm^2^) and $\overline{\boldsymbol{SI}}$ equals the mean SI of all three b-values at the given ROI position.
- ADC_4: *Dedicated task card on post-processing workstation* (MMWP: MultiModality Workplace, software version B19, Siemens Healthcare Division). According to prior exploratory analysis [10], noise reduction was set to a level of 10 (arbitrary units of pixel intensities), which generated a visual aspect similar to that of ADC_1.

The calculations of ADC_2 and ADC_3 were performed in Excel (v 15.16, Microsoft Corp., Redmond, WA, USA) on Mac OS 10 (Apple Inc., Cupertino, Ca). Further details on the ADC calculation are listed in the Supplementary Material section.

### Assessment of lesions

DICOM files of raw DWI data and the two parametric ADC maps (ADC_1, ADC_4) were imported on a MMWP. Previous investigations have verified observer-related bias for the assessment of ADC values (CV from 6.8 to 7.9 [6]). As our study focused on the impact of PPMs on the ADC value, a single-read and single-reader approach was chosen to decrease such potential reader-dependent bias. First, lesions had to be identified based on the following criteria:

- *Definition*: A lesion was defined as a malignant focus (primary, organ metastasis or lymphoma, c.f. Table 1). Lesions were identified on raw DWI data based on the SOR.
- *Lesion size*: In order not to be biased by partial volume effects, a minimum lesion diameter of 1.5 cm was defined and the lesion had to be clearly visible in three consecutive slices on DWI images and ADC maps.
- *Multiple lesions per patient*: If multiple lesions were present within one patient, only one lesion was included in every anatomic region (e.g. cervical, mediastinal, retroperitoneal lymph nodes (LN)). The maximum numbers of lesions assessed per patient was set to seven. This was done so as not to overload the study collective by data from patients at advanced disease stages.

Second, lesions were assessed by regions of interest (ROIs). The latter were defined according to the following criteria:

- *Positioning*: One circular ROI was carefully positioned in order to encircle a representative area of the lesion with restricted diffusion.
- *Size*: In order to minimize partial volume effects, the target ROI size was set to 1 cm^2^.
- *Transfer of ROI coordinates between PPMs*: In previous works on the reproducibility of the ADC, ROI coordinates between different workstations were transferred manually [6, 11]. This approach is prone to a user-dependent bias. Our approach used user-independent software for this purpose (MMWP). It enables the automatic transfer of slice number, size and centre of the circular ROI between each PPM and the raw DWI data. Accordingly, user-dependent bias can be excluded and differences between ADC values of the PPM are related only to the underlying algorithms of the PPM.

**Fig. 1:**
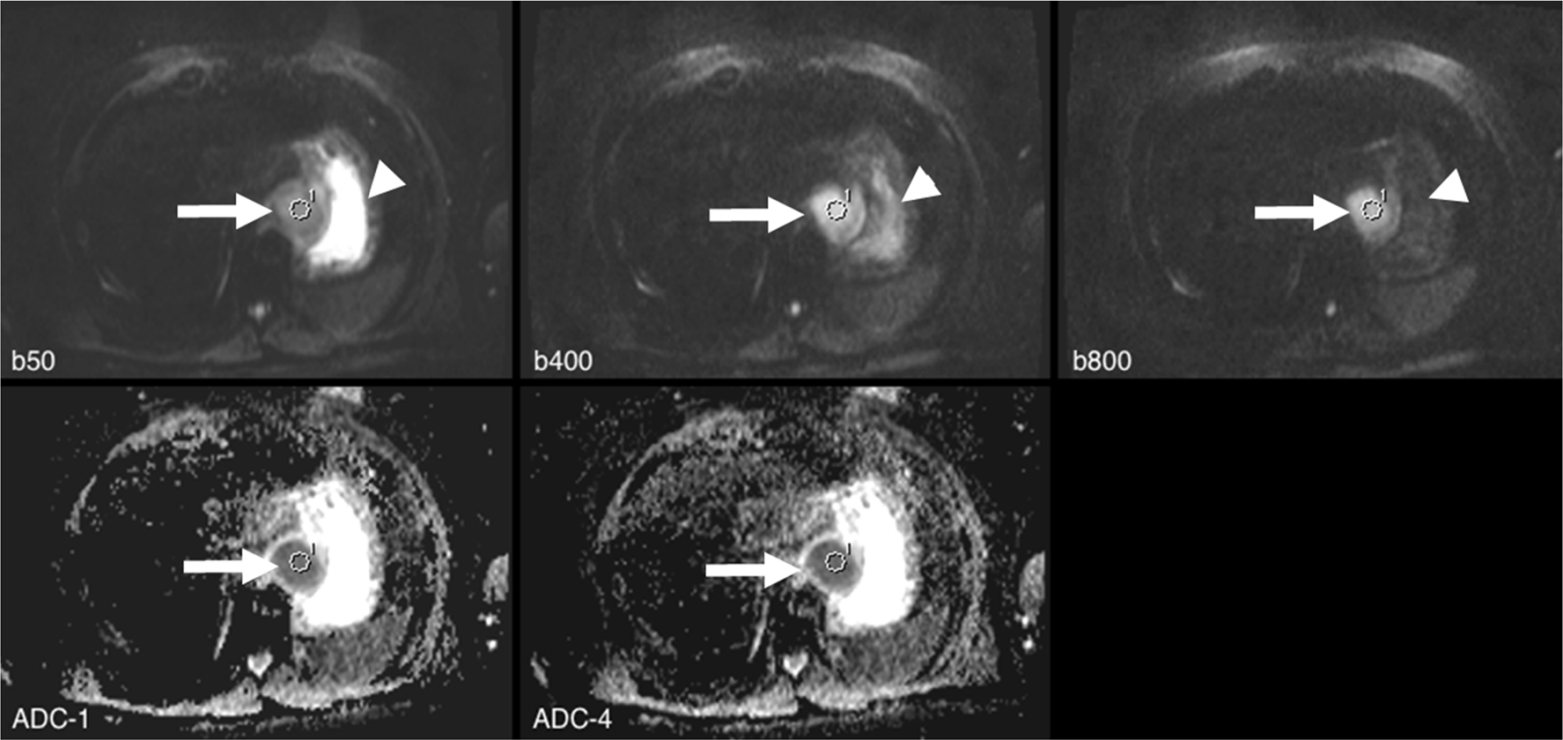
Examples of reading approaches: A 55-year-old male with advanced-stage oesophageal cancer (arrow). The image shows the reading set-up, with raw diffusion-weighted imaging (DWI) data on the top row (b50, b400, b800 [s/ mm^2^]), and, below, parametric apparent diffusion coefficient (ADC) maps of ADC_1 and ADC_4. The latter were automatically generated inline by the scanner or by the viewing workstation (MMWP, details see above). On raw DWI data, the typical signal decay of free water can be depicted in the adjacent stomach, whereas the lesion itself shows signal alteration indicative of diffusion restriction (arrowhead). The corresponding ROI 1 was placed within the tumour in the b800 image and ROI coordinates were automatically transferred to all other remaining series. This approach excluded user-dependent bias when comparing the ROI statistics between the given series

**Fig. 2:**
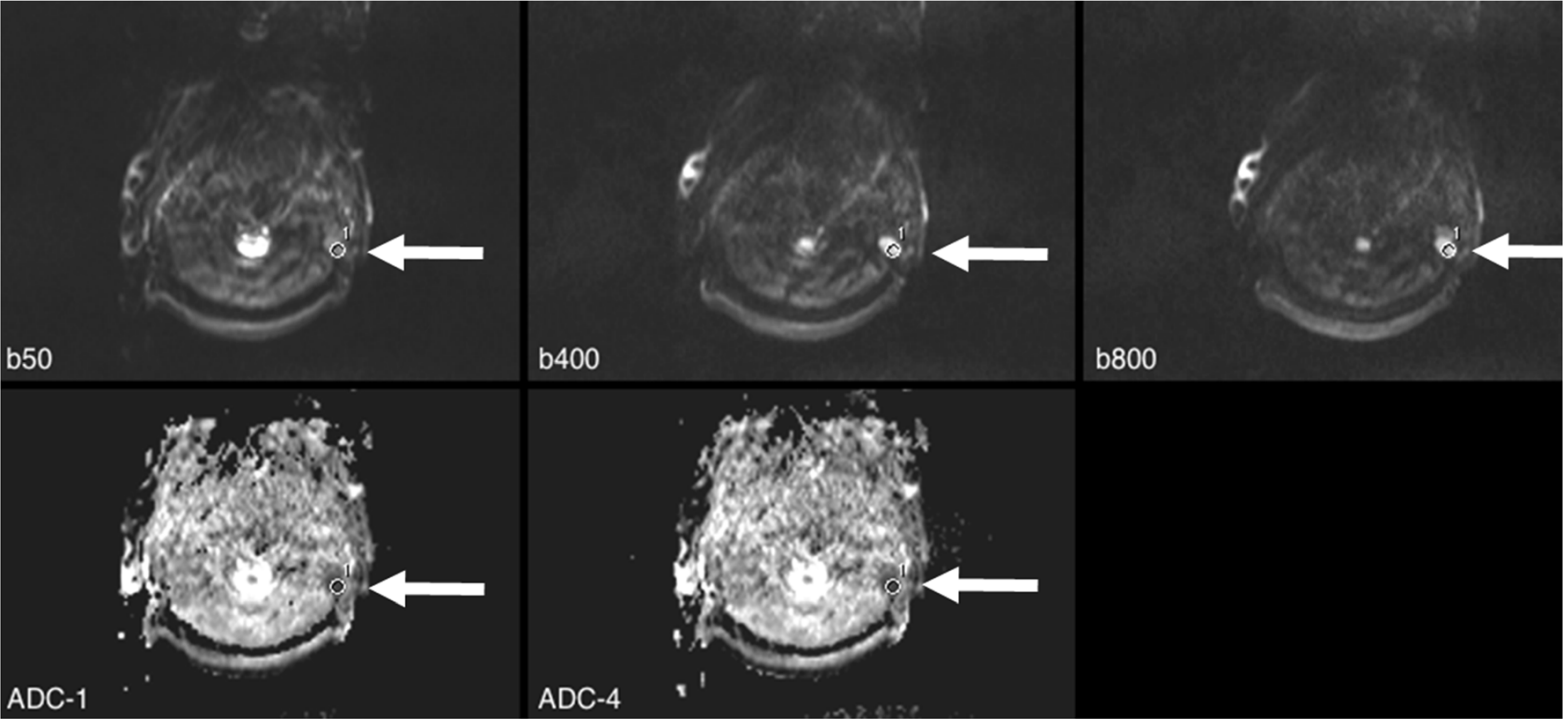
A 59-year-old male with advanced-stage lung cancer and multiple cervical lymph node metastases. Set-up and automatic region of interest (ROI) transfer were the same as that in Fig. 1. A signal-to-noise (SNR) of 21.9 at b800 [s/mm^2^] was achieved in this small lesion that showed diffusion restriction as follows: apparent diffusion coefficient (ADC) values ranged from 0.453 (ADC_1) to 0.458 (ADC_4). Using the formulas (1) and (2) (see text), slightly higher values were calculated (ADC_2 = 0.459 and ADC_4 = 0.464)

**Fig. 3:**
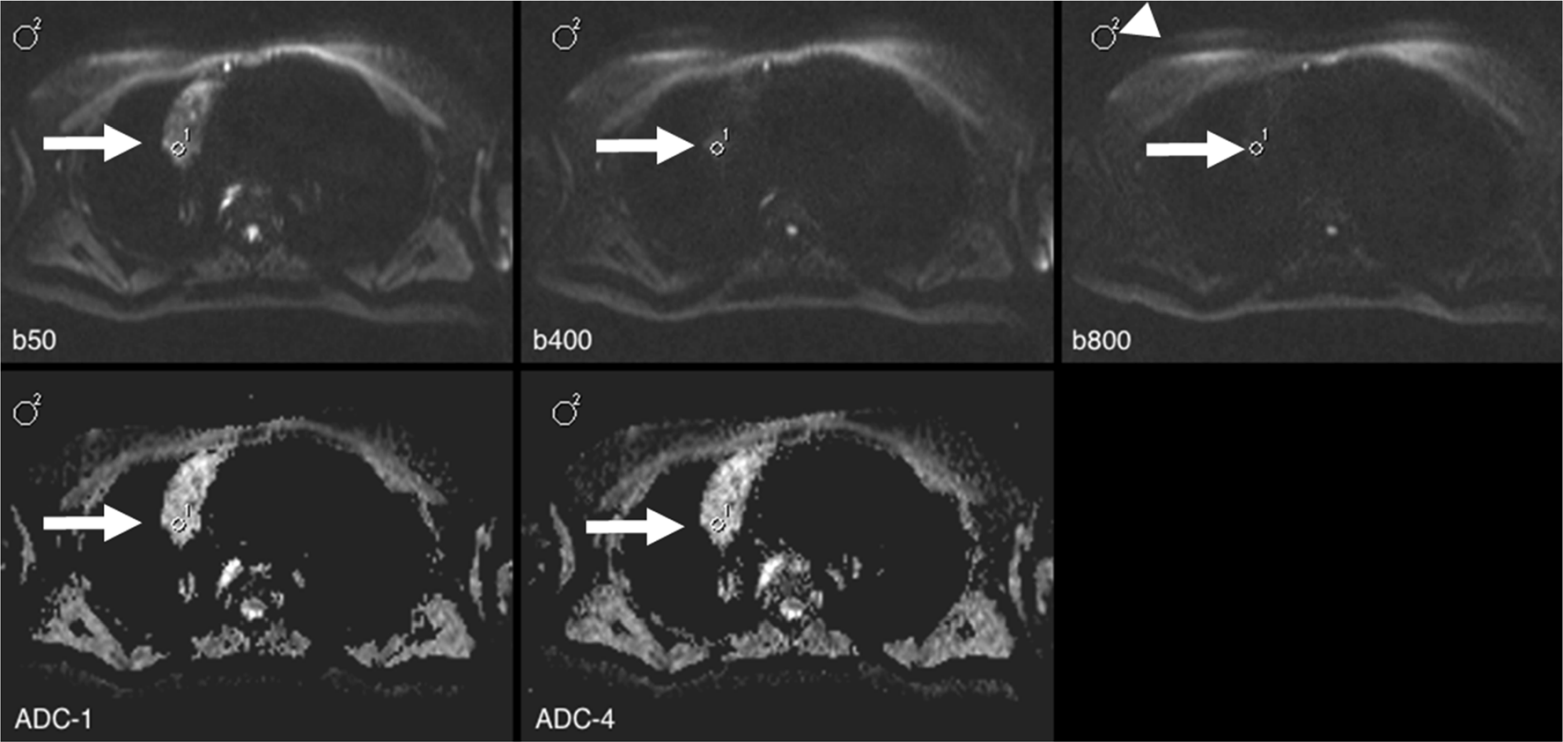
A 47-year-old female diagnosed with Hodgkin’s lymphoma. Protocol was identical to that in Fig. 1. In this case, the corresponding region of interest (ROI) 1 (arrow) was placed within the tumour (noise ROI: arrowhead). Apparent diffusion coefficient (ADC) values ranged from 1.800 (ADC_1) to 1.972 (ADC_2; ∆ = 9 %). Note the low remaining signal at b800 (signal-to-noise ratio (SNR) of 3.5) in this heterogeneous lesion

This reading workflow is demonstrated on three clinical examples in Figs. 1, 2, and 3. Finally, the mean value of SI (raw DWI data) and the ADC (ADC_1 to ADC_4) of each lesion ROI was documented in a central Excel database.

### Statistical methods

Data analysis followed a lesion-based approach and the independence of lesions in the same patient was assumed.

We evaluated the distribution of ADC values within each PPM (ADC_1 to ADC_4) and performed pairwise comparisons of the PPM (i.e. ADC_1 vs. ADC_2, ADC_1 vs. ADC_3, etc.).

Descriptive data analysis included arithmetic mean, relative difference (∆), median, SD (standard deviation), range (minimum to maximum), percentiles (5, 25, 75, 95) and the coefficient of variation (CV [%] = 100*SD/mean; [6, 11]).

ADC values were not normally distributed, as shown by the D’Agostino-Pearson test (*P* < 0.05), with differing means and medians, as well as visual analysis (box plots). Thus, pairwise comparison of the four PPMs was obtained using the Wilcoxon signed-rank test (α = 1 %). *P*-values are given uncorrected, but results were interpreted considering potential alpha error.

Visual analysis was performed using box plots and Bland-Altman plots (BAPs). BAPs were used to check for systematic and proportional error between the four PPMs on the level of pairwise comparison. ∆PPM (PPM_1 minus PPM_2) was placed on the ordinate and PPM_1 on the abscissa. A regression line was placed into the point cloud of each BAP. If it the regression line could be fitted to the point cloud (criterion: slope, intercept: P < 0.05), the presence of a proportional error was assumed [12].

Statistical analyses were performed using MedCalc for Windows, version 12.5 (MedCalc Software, Ostend, Belgium).

## Results

Mean ADC values of the PPMs ranged from 1.136 (ADC_1) to 1.206 (ADC_3; Table 3). This led to a relative ADC difference ∆ of up to 7.0 %.

**Table 3:** Descriptive data analysis of apparent diffusion coefficient (ADC)-values in four post-processing methods (PPMs)

PPM	Mean	SD	Median	Range	25–75 P	5 - 95 P
ADC_1	1.136	0.435	1.034	0.312–2.540	0.871–1.352	0.478–2.002
ADC_2	1.201	0.473	1.057	0.313–2.744	0.905–1.457	0.614–2.152
ADC_3	1.206	0.473	1.062	0.315–2.763	0.904–1.473	0.643–2.146
ADC_4	1.163	0.438	1.044	0.317–2.599	0.894–1.389	0.607–2.030

All values given in [10^-3^ mm^2^/s]

*SD* standard deviation, *P* percentile

**Fig. 4:**
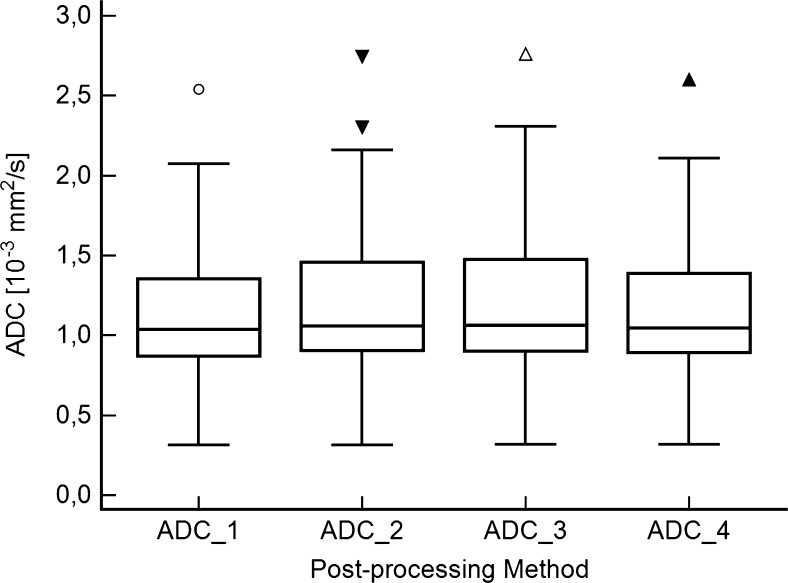
Box plots summarizing the distribution of apparent diffusion coefficient (ADC) values provided by the four different post-processing methods (PPMs). Median ranks between the PPMs showed significant differences (P ≤ 0.003). Note the different size of boxes and whiskers at the upper range of ADC values. In ADC_2, there were two outliers beyond the 97.5 quartile. One bone metastasis of lung cancer (ADC = 2.301) and one prostatic metastasis of a neuroendocrine tumour (ADC = 2.744) are shown. The latter caused all the outliers in the remaining PPMs

With a ∆ = 8 %, dispersion of data was pronounced in the upper range of ADC values (Fig. 4). Thus, maximal values reached from 2.540 (ADC_1) to 2.763 (ADC_3). As shown in Fig. 4, comparable results were observed for the 95th percentiles (2.002: ADC_1 to 2.152: ADC_2). On the lower end of ADC, data were less scattered. Minimum values ranged from 0.312 (ADC_1) to 0.317 (ADC_4), with ∆ ≤ 1.6 %.

The pairwise comparison of ADC values revealed mean differences of ADC values ranging between −0.070 (ADC_1 vs. ADC_3) and 0.043 (ADC_3 vs. ADC_4; Table 4). On a case-by-case basis, such differences reached up to −0.866 (maximum difference for ADC_2 vs. ADC_4) or −0.137 in case of the 25th–75th percentile (ADC_1 vs. ADC_3).

**Table 4:** Comparison of post-processing methods (PPMs): Descriptive data analysis

PPM-pair	Mean	SD	Range	25–75 P
(1) ADC 1 vs. ADC_2	-0.065	0.157	-0.483 to 0.81	-0.128 to -0.012
(2) ADC_1 vs. ADC_3	-0.070	0.159	-0.493 to 0.806	-0.137 to -0.015
(3) ADC_1 vs. ADC_4	-0.027	0.046	-0.36 to 0.006	-0.032 to -0.007
(4) ADC_2 vs. ADC_3	-0.005	0.019	-0.14 to 0.013	-0.008 to 0.002
(5) ADC_2 vs. ADC_4	0.038	0.146	-0.866 to 0.454	0.005 to 0.092
(6) ADC_3 vs. ADC_4	0.043	0.148	-0.861 to 0.465	0.002 to 0.106

*Note* Mean: Given are relative mean differences between each PPM-pair (e.g. of ADC_1 minus ADC_2). Corresponding parameters of descriptive statistics are given: Difference: All ADC-Values given in [10^-3^ mm^2^/s]. Negative values indicate that the first PPM provided smaller values than the second one (e.g. in Pair (1) on average ADC_1 values were lower by 0.065 10^-3^ mm^2^/s compared to ADC_2)

All values given in [10^-3^ mm^2^/s]

*ADC* apparent diffusion coefficient, *SD* standard deviation, *Range* from minimum to maximum difference, *P* percentile

Significant differences between all PPMs were noted (ADC_2 vs. ADC_3: *P* = 0,003, all other pairs: *P* < 0.001; c.f. Table 5). This led to a CV between 1.1 % (ADC_2 vs. ADC_3) and 10.4 % (ADC_1 vs. ADC_3). This gave a mean CV of 7.2 % (8.4 % if ADC_2 was not considered).

**Table 5:** Differences between post-processing methods (PPMs): Detailed analysis

PPM-pair	P	Slope	Intercept	Outliers	CV
(1) ADC_1 vs. ADC_2	<0.0001	n.s.	n.s.	4	10.2
(2) ADC_1 vs. ADC_3	<0.0001	n.s.	n.s.	4	10.4
(3) ADC_1 vs. ADC_4	<0.0001	n.s.	n.s.	1	3.2
(4) ADC_2 vs. ADC_3	0.003	n.s.	n.s.	1	1.1
(5) ADC_2 vs. ADC_4	<0.0001	0.12	-0.11	3	9
(6) ADC_3 vs. ADC_4	<0.0001	0.13	-0.1	3	9.1

*Note* P value according to Wilcoxon signed-rank test

**Fig. 5:**
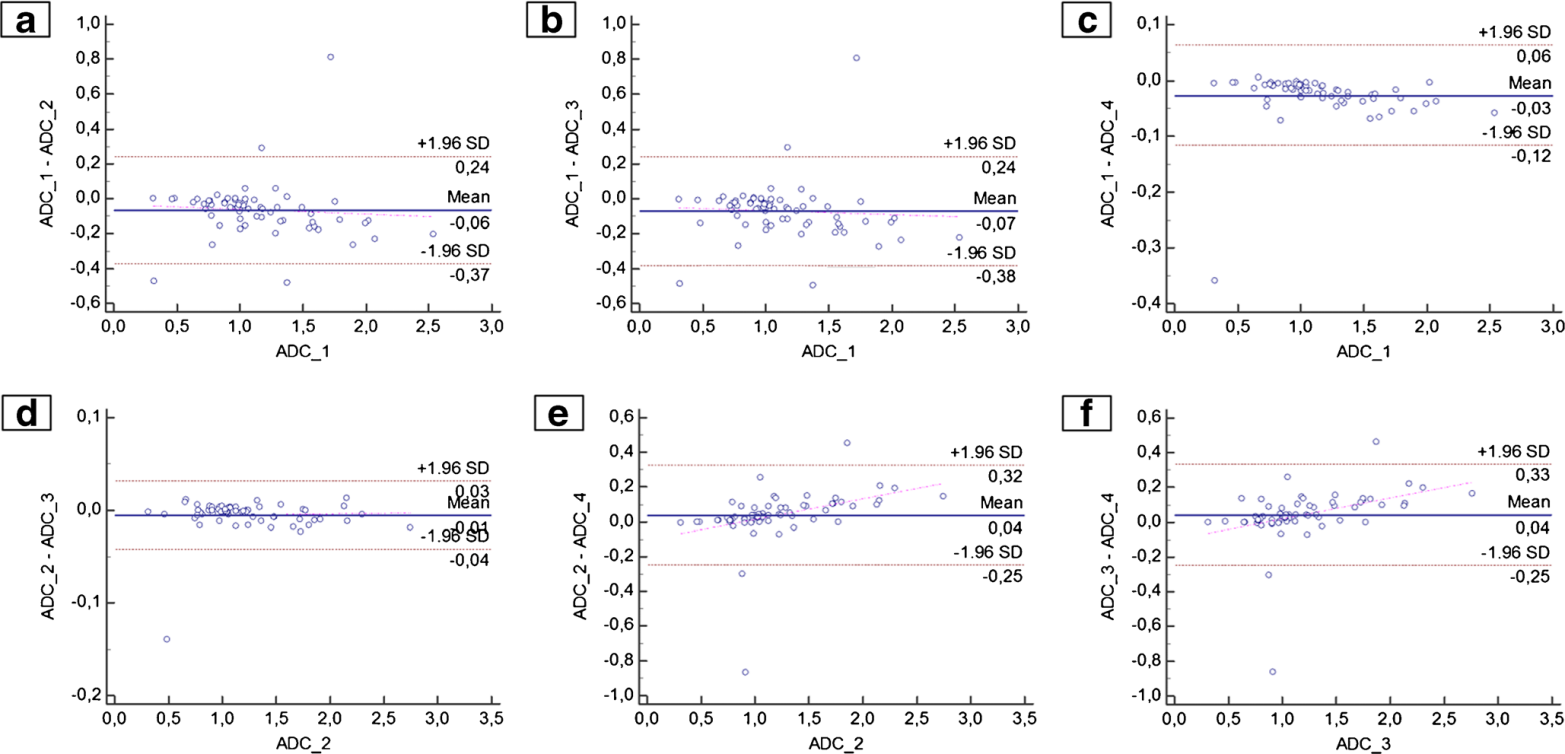
Pairwise comparison of post-processing methods (PPMs) using Bland-Altman plots (BAPs). ∆PPM (PPM_1 minus PPM_2) is shown on the *ordinate*. PPM_1 is shown on the *abscissa*. Indicated are the limits of agreement, the mean relative difference, and the regression curve. Note the significant difference in mean ADC value in each pair as well as the presence of up to four outliers beyond the limits of agreement (+/- 1.96*SD: **A** and **B**). A proportional error was identified for **E** and **F**, with a slope of 0.12 and 0.13, respectively

If there was a significant (P < 0.05) correlation between x (PPM_A) and y (PPM_A minus PPM_B), the slope and intercept of the corresponding regression equation are given. These results indicate a proportional error within PPM-pairs (5) and (6); see also Fig. 5. Accordingly, the difference between ADC_2/ADC_3 and ADC_4 increased with rising ADC-levels

*ADC* apparent diffusion coefficient, *Outliers* number of ADC-values beyond +/-*1.96* * *SD, CV* coefficient of variation (%)

Visual analysis of BAP (Fig. 5) excluded the presence of systematic error. However, up to four outliers (ADC_1 vs. ADC_2, ADC_1 vs. ADC_3) were noted beyond the levels of agreement. Only one outlier was noted in two PPM pairs (ADC_1 vs. ADC_4 and ADC_2 vs. ADC_3).

A proportional error was identified in two PPM pairs (ADC_2 vs. ADC_4 and ADC_3 vs. ADC_4). Accordingly, differences between such pairs were significantly correlated (P < 0.05) with the magnitude of measurements. Namely, differences increased with rising ADC levels (slope = 0.12: ADC_2 vs. ADC_4; slope = 0.13: ADC_3 vs. ADC_4).

## Discussion

DWI is an essential part of state-of-the-art oncological MR protocols. One reason for the unique success of DWI is certainly the seemingly easy way to interpret ADC maps. Concurring techniques – such as MR spectroscopy – require far more sophisticated post-processing, whereas ADC maps are usually generated fully automatically inline by the scanner.

In the literature, there are few clinical reports on the variability of the ADC. Essentially, there are three aspects that should be addressed in order to investigate the variability of the ADC:

First, ADC is influenced by the *imaging protocol* itself. Thus, many factors must be considered. Changing the echo time (TE), numbers of averages, spatial resolution or size of the field of view (FOV), etc., will have an impact on the signal-to-noise ratio (SNR). The latter plays a key role in the generation of raw DWI data and has an important impact on ADC values [2, 13]. However, factors such as the scanner itself, sequence type, coils and vendors are also likely to have an impact on ADC values. Due to the number of influencing factors, it is difficult to express the effect of the imaging protocol itself on final ADC values in a simple number.

Corona-Villalobos et al. [11] performed serial measurements both of healthy tissue and a phantom using two different DWI sequences. The variability of corresponding ADC values were analyzed and quantified by a mean CV of 11 %. Donati et al. [14] compared ADC values of healthy volunteers within various regions of the abdomen. They used six different scanners sold by three different vendors at 1.5 and 3 Tesla field-strength. Those authors reported significant inter-vendor differences, with a minor effect of field strength. CV ranged from 7.0 % to 15.9 % if the liver ROIs were not considered. Of note, the CV of liver lesions was much higher (up to 27.1 %).

Second, identification of the ADC values depends on the *radiologist her-/himself*. This means that ADC assessment – although a quantitative measure by nature – is influenced by observer-related bias. This fact is due to inter- and intra-observer variability regarding manual ROI placement by the reader. A paper recently published by Clausner et al. [6] focused on this particular aspect of ADC analysis. The authors quantified this observer-related bias with a mean CV of 7.2 % (range 6.8–7.9 %). This value is in the range of ADC variability caused by the PPMs, according to our results (CV: 7.2).

Third, the *PPMs* of DWI data might have an impact on ADC values. Different from the first two, this fact has been largely ignored by the radiological community. Basic and computational scientists have developed a variety of different algorithms to calculate the ADC based on raw DWI data. All such approaches work slightly differently, and, thus, are likely to generate different numerical values. Of note, many software solutions being used in clinical, as well as scientific practice, are basically black boxes, as PPMs for DWI data are not generally available to the user. Based on an oncological dataset, we intra-individually compared ADC values of four different PPMs typically used for this purpose.

In our series, average ADC values did show a range of 7.0 %, providing values between 1.136 and 1.206. As these two extremes were calculated by the proprietary scanner software (ADC_1: the exact algorithm is not disclosed) and the ordinary least squares fit (ADC_3), results are indicative of further post-processing in the former. This could include fitting and smoothing algorithms, as well as the filtering of raw data. We did not aim to identify the best algorithm for the calculation of the ADC, yet, from a scientific perspective, the use of a black box tool should be discussed critically (ADC_1), particularly if the results differ significantly from an open-source tool such as that used for method ADC_3. However, average values showed not only significant differences between the two extremes, but also between all other methods (all pairwise comparisons: *P* ≤ 0.003).

One should question whether *statistical significance* really translates into *clinical relevance*. One approach to the interpretation of ADC maps in clinical practice is visual inspection. If such a qualitative analysis of ADC maps is the task, the choice of different post-processing algorithms certainly has a minor impact on final radiological assessment. However, if quantitative measurement is performed, the reader should be aware of this potential bias. This is becoming increasingly important, because a growing number of scientific papers suggest definitive ADC thresholds for differential diagnosis.

In a recent article, Baltzer et al. [15] proposed an ADC threshold of 1.4 to differentiate benign from malignant *breast* lesions. Data was supported by good specificity (80.5 %) and sensitivity (100 %), which was improved by integrating contrast-enhanced MRI (specificity 96.1 %, sensitivity 100 %). The authors used ADC maps that were automatically generated by the scanner software. Noise reduction level was set to an arbitrary level of 30 [15].

Similarly, the ADC was reported as a promising tool for differentiating focal *liver* lesions as benign or malignant. For example, ADC values under 1.470–1.600 were described as a potential sign of malignancy again with good, yet varying sensitivity (74–100 %) and specificity (77–100 %) [16–22]. Kim et al. [18] reported the use of a linear logarithmic regression. The other authors measured the ADC using ROIs on ADC maps.

Recently, DWI has become a popular tool for MR phenotyping of *prostate* lesions. Indeed, ADC could be used to predict Gleason grades, to stratify into further treatment groups (watchful waiting vs. therapeutic intervention), and to assess treatment response [23–25]. Again, methodological documentation within such papers on DWI post-processing is sparse, and the authors reported the use of only ADC maps that were generated by the scanner software [23–25].

Up to this point, we have discussed our results in the context of mean values provided by the four DWI PPMs. This approach averages out a number of details that are important for clinical practice. For example, mean values of ‘method A’ might be exactly the same as of ‘method B’. However, ‘method A’ might still produce different results on a pairwise comparison in certain cases. In fact, this is exactly what we observed in our data. Such details are of clinical importance and should be discussed.

As summarized in Table 3 and Fig. 4, variances between PPMs were pronounced in the upper range of ADC values (maximum: 2.540–2.763, ∆ = 8 %). The highest values were generated by ADC_2 (up to 2.744) and ADC_3 (up to 2.763). In comparison, the maximum ADC values generated by the proprietary algorithms were lower (ADC_1: 2.540, ADC_4: 2.599). However, dispersion of data was much smaller at the lower range of ADC values. Minimum values ranged from 0.312 (ADC_1) to 0.317 (ADC_4), giving a ∆ ≤ 1.6 %. Such a finding could be due to low SNR on the b800 images [2].

Differences were noted not only at the extremes, but also in terms of data distribution. This is reflected by a mean CV of 7.2 %. As shown in Table 4, differences also reached up to 0.137 in the 25th–75th percentile (ADC_1 vs. ADC_3). According to the point clouds of the BAP (Fig. 5, Table 5), proportional error could be identified between the ADC_4 and both open-source algorithms (ADC_2 and ADC_3; Fig. 5 E, F). Accordingly, the difference between such PPMs increased with the rising magnitude of ADC values.

Our results are of clinical importance. As the widespread clinical application of quantitative DWI is continuously increasing, academic MR radiologists are not the only group that should be aware of the impact of PPMs on ADC values. This effect might be relevant even within one single institution. For instance, if dedicated post-processing methods are used in addition to the standard ADC maps provided by the MR system, ADC metrics might be different. Therefore, we recommend the standardization of PPMs. This is of the utmost importance in longitudinal studies, for example, during follow-up of chemotherapy, in order to evaluate treatment response [3].

## Limitations

In addition to the PPMs, in the present analysis, all other ‘confounding factors’ on ADC estimates were set constant. This approach was required to determine the exact effect of PPMs on ADC metrics. Accordingly, the results of our WB DWI study cannot be translated into other clinical scenarios literally. Such other ‘confounding factors’ are likely to further increase the variability of ADC-metrics in addition to the effect of PPMs. This is why they should be discussed briefly.

*First*, ADC metrics depend on the *imaging protocol* itself. It is well known that the protocol is not constant, but has to be optimized for the specific scenario. For instance, if a dedicated examination of the upper abdomen is required, parameters will necessarily differ from our protocol. For instance, more b-values will be chosen in this case [14], whereas a dedicated breast MRI [15] or even a WB DWI protocol will require different settings [3].

Yet, even in the given WB imaging scenario, different protocols coexist. Accordingly, some research teams favour the use of high b-values for this purpose, and skip low values below 200 for WB MRI [13]. In this scenario, the ADC value is, again, likely to be different from our data.

Future investigations should assess to what degree differences between PPMs are present, if DWI protocols are altered. Special attention should be paid to the comparison of ADC values derived only from the high-b-value signal intensities.

*Second*, there is no single *way to document the results of ROI* measurement. As the latter sums up the ADC values of every pixel within the given ROI, many metrics can be used for this purpose. These include *minimum*-ADC, *maximum*-ADC or *histogram analysis*. However, in clinical practice, the mean ADC value within the ROI is typically used [15]. This is why we chose this approach.

If the method of ROI analysis is changed, differences between the PPMs might also be altered. This is likely if a pixel-by-pixel comparison is performed between ADC metrics derived from various PPMs. As this approach is particularly capable of highlighting outlier values, it should be investigated in future studies.

*Third*, *repeatability* of DWI measurements is a limitation in itself. Thus, during serial measurements of a given pathology, ADC values will not be constant and will necessarily scatter. Even if all other ‘confounding factors’ – including the PPM itself – are set as constant, the repeatability ADC values will not be perfect. This effect has been reported by [11].

Certainly, such considerations limit the literal translation of our results into clinical practice. However, we did not aim to establish ‘the optimal PPM’. In fact, the aim of our study was to demonstrate that “PPM has an impact on ADC values”. Even if absolute differences between PPMs change due to altered study protocols, this key point will certainly hold true.

## Conclusion

Post-processing of raw DWI data and calculation of the ADC is a delicate act and depends on the choice of the post-processing algorithms. We observed significantly different mean ADC values between all of the four algorithms tested, and demonstrated substantial intra-individual differences on a case-by-case basis, leading to a mean CV of 7.2 %. As the widespread clinical application of quantitative DWI is constantly increasing, MR radiologists should be aware of this phenomenon.

## Electronic supplementary material

Caption of the data object (PDF 466 kb)

## Footnotes

1 In the following parts of the manuscript, the unit of the ADC given as [10^-3^ mm^2^/s] will be omitted to improve legibility.
