# Supplementary material for: Impact of post-processing methods on apparent diffusion coefficient values
Source: Eur Radiol. 2016 Jun 1;27(3):946–55. doi: 10.1007/s00330-016-4403-6 (PMC5591618; doi:10.1007/s00330-016-4403-6)
Supplement: Supplementary file 1 — Caption of the data object (PDF 466 kb) [file 330_2016_4403_MOESM1_ESM.pdf]

Supplementary material

Content

ADC calculation **1**

The choice of the b-values **2**

Exploratory subgroup analysis **4**

Is there an influence, if patient based analysis (PBA) is favored instead of lesion based analysis (LBA)? 5

Is there an influence, if lesions are stratified by pathology? 8

References.. 15

ADC calculation

The degree of signal attenuation depends on multiple factors as shown in [1]

[I] $SI = SI_0 * e^{-b*D}$

SI_0 = signal intensity of the T2-weighted image without applied gradient pulse

b = b value: degree of diffusion weighting [s/mm^2], depending on gradient amplitude, duration and temporal spacing of the two motion-probing gradients

D = diffusion coefficient

With a set of two different b values the formula can be applied as follows[2]:

[II] $ADC = \frac{ln\frac{SI_1}{SI_0}}{b_0 - b_1}$ [mm^2/s]

This formula demonstrates that in a two-point technique the ADC represents the slope of a semi-logarithmic curve which is obtained when the logarithm of relative signal intensity in a tissue is plotted along the y-axis and b values along the x-axis [3].

In the case of more b values, other techniques to determine a regression curve have to be performed. In the simplest case, a linear relationship between the variables is supposed. One example is the ordinary (linear) least squares estimation[4,5]: "Least squares" means that the method is based on the principle to keep as low as possible the sum of the squares of the errors i.e. the distance from the calculated regression curve.
The regression coefficient b_{yx} is calculated as follows [6]:

$$[\text{III}] \quad b_{xy} = \frac{\sum_{i=1}^{n}\left((x_i-\bar{x})*(\mathrm{y_i}-\bar{y})\right)}{\sum_{i=1}^{n}(x_i-\bar{x})^2}$$

- x_i, y_i = variables
- $\bar{x}$, $\bar{y}$ = arithmetic mean of x resp. y

So, in a set of three b values (e.g. 50, 400, 800 s/mm^2), the equation would be:

$$(\text{IV})\ ADC = \frac{(b_{50}-\bar{b})*(\ln SI_{50}-\overline{\ln SI})+(b_{400}-\bar{b})*(\ln SI_{400}-\overline{\ln SI})+(b_{800}-\bar{b})*(\ln SI_{800}-\overline{\ln SI})}{(b_{50}-\bar{b})^2+(b_{400}-\bar{b})^2+(b_{800}-\bar{b})^2}$$

The choice of the b-values

It has been postulated that in sets of three b-values, if the third b-value (e.g. 0, 400, 800 s/mm^2 or 0, 500, 1000 s/mm^2) is a multiple of the second, the latter should have no impact on the line slope, i.e. the ADC[7]. Regarding at

[I] $ADC = \frac{(b_{50} - \bar{b}) * (\ln SI_{50} - \overline{\ln SI}) + (b_{400} - \bar{b}) * (\ln SI_{400} - \overline{\ln SI}) + (b_{800} - \bar{b}) * (\ln SI_{800} - \overline{\ln SI})}{(b_{50} - \bar{b})^2 + (b_{400} - \bar{b})^2 + (b_{800} - \bar{b})^2}$

this would signify, that

[II] $(b_{400} - \bar{b}) * (\ln SI_{400} - \overline{\ln SI}) = 0$

or simpler

[III] $b_{400} - \bar{b} = 0$

This demonstrates that if the middle b-value represents the arithmetic mean of the three b-values it actually has no impact on the ADC.
Thus we can calculate:

[IV] $ADC = \frac{(b_{50} - \bar{b}) * (\ln SI_{50} - \overline{\ln SI}) + (b_{800} - \bar{b}) * (\ln SI_{800} - \overline{\ln SI})}{(b_{50} - \bar{b})^2 + (b_{800} - \bar{b})^2}$

In this case, mathematical simplification of (IV)

Considering

[V] $\overline{\ln SI} = \frac{\ln SI_{50} + \ln SI_{800}}{2}$; $\bar{b} = \frac{b_{50} + b_{800}}{2}$; $\ln SI_{50} - \ln SI_{800} = \ln \frac{SI_{50}}{SI_{800}}$

leads to the original two-point formula

[VI] $ADC = \frac{\ln \frac{SI_{50}}{SI_{800}}}{b_{50} - b_{800}}$

In our set of b-values 50, 400 and 800 s/mm^2 the arithmetic mean $\bar{b}$ = 416,67 differs slightly from 400.

Exploratory subgroup analysis

Is there an influence, if patient based analysis (PBA) is favored instead of lesion based analysis (LBA)?

Method:

- **PBA**: Definition of a new subgroup. Only one lesion per patient.
  - Patients with single lesions: Left unchanged
  - Patient with multiple lesions: Only one lesion included into analysis. Lesion selection as follows:
    - If primary lesion present (bronchial cancer …), selection of the primary lesion and skipping of metastatic foci.
    - If no primary lesion evident *random* selection of one index lesion.
- Comparison of ADC metrics within each group (PBA and LBA): Friedman test for dependent samples, if test was P<0,05 → pairwise comparison of subgroups according to Conover was performed [8].

Results:

The results of the whole study collective (LBA) can be reproduced in PBA were alike:

Variable	Different (P<0,05) from variable*	Comment
(1) ADC_1	(2) (3) (4)	ADC_1 gives the lowest values
(2) ADC_2	(1) (4)	ADC_2 and ADC_3:<br>• give the highest values<br>• are similar (n.s.)
(3) ADC_3	(1) (4)	
(4) ADC_4	(1) (2) (3)	ADC_4 gives intermediate values

*for PBA and LBA

Conclusion:

Results are not biased by the level of analysis. The same results are achieved for PBA and LBA.

Details:

PBA

Variable	Different (P<0,05) from variable nr
(1) ADC_1	(2) (3) (4)
(2) ADC_2	(1) (4)
(3) ADC_3	(1) (4)
(4) ADC_4	(1) (2) (3)

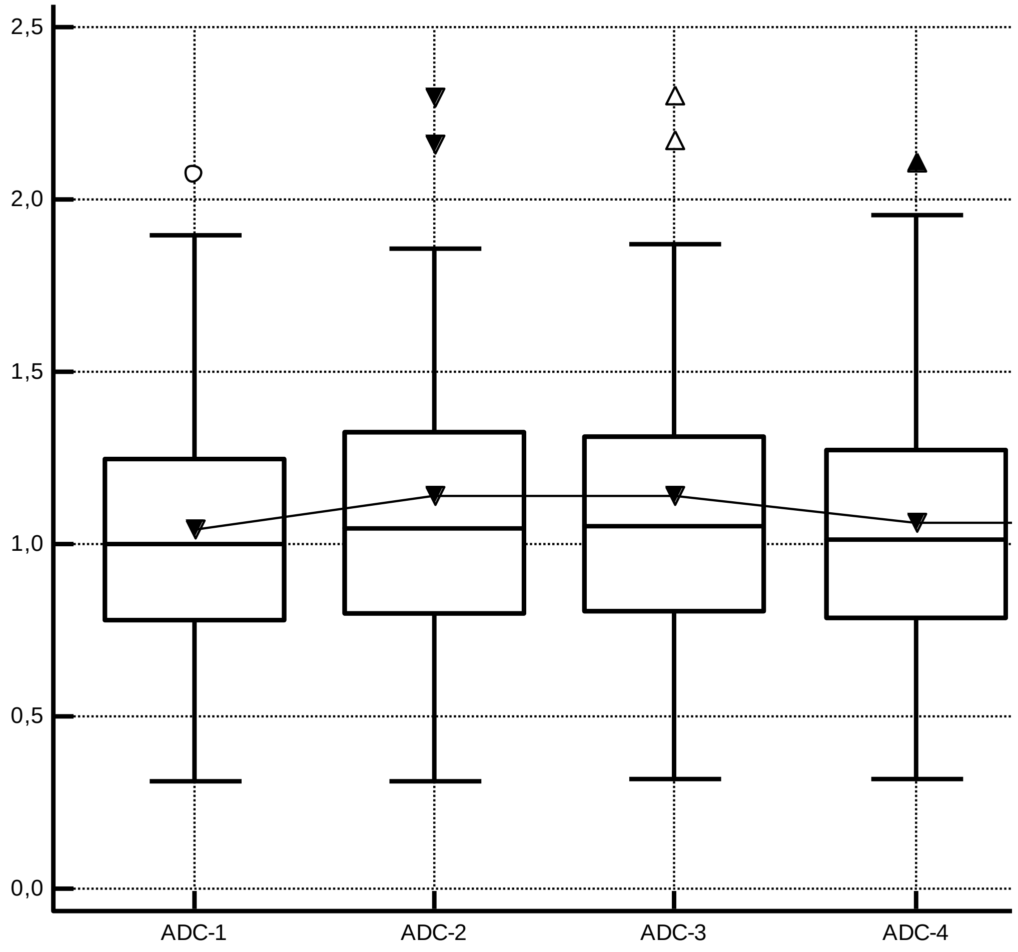

Figure 1: PBA – ADC values stratified by PPM. ADC (y-axis) are given in [10^{-3} mm^2/s].

LBA

Variable	Different (P<0,05) from variable nr
(1) ADC_1	(2) (3) (4)
(2) ADC_2	(1) (4)
(3) ADC_3	(1) (4)
(4) ADC_4	(1) (2) (3)

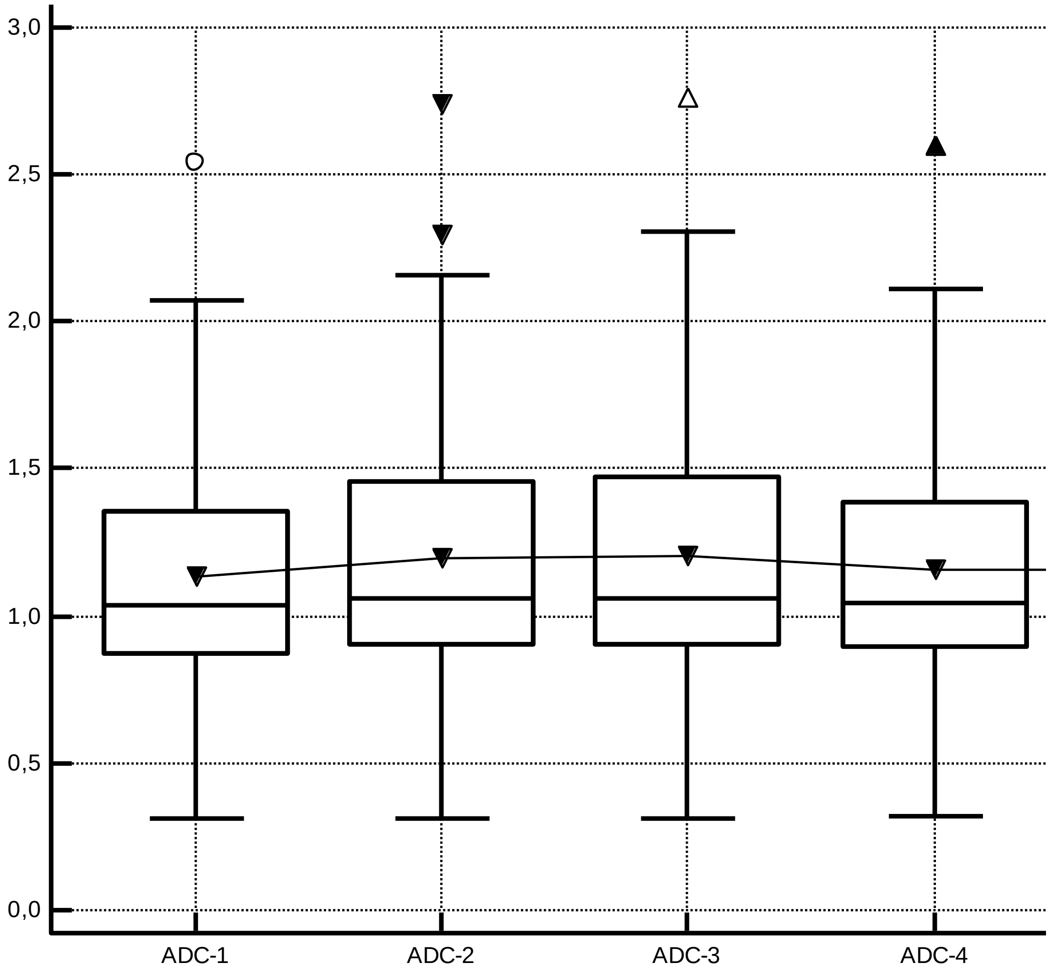

Figure 2: LBA – ADC-values stratified by PPM. ADC (y-axis) are given in [10^{-3} mm^2/s].

Is there an influence, if lesions are stratified by pathology?

Method:

- (I) Comparison of pathology (**LK**: lesion within lymph nodes. **Wo_LN**: non-lymph node lesion) for each ADC metric by Mann-Whitney test for independent samples. For example
  - ADC_1 lymph node case only vs. ADC_1 non lymph node cases
  - ADC_2 lymph node case only vs. ADC_2 non lymph node cases
  - Etc.
- (II) Comparison of ADC metrics (ADC_1, ADC_2 etc.) within each pathology (given as lymph node vs. no lymph node. Stats: Friedman test for dependent samples, if test was P<0,05 pairwise comparison of subgroups according to Conover was performed [8]. For example:
  - Lymph node case only: ADC_1 vs ADC_2 vs ADC_3 vs. ADC_4
  - Non lymph node cases: ADC_1 vs ADC_2 vs ADC_3 vs. ADC_4

Results:

(I) Pairwise comparison of ADC metrics revealed at best only minor and non-significant differences for all methods: ADC_1 (P = 0,68), ADC_2 (P = 0,93), ADC_3 (P = 0,91), ADC_4 (P = 0,60) and ADC_MWPP (P = 0,60).

(II) Differences of ADC metrics known from the whole patient collective could be reproduced in both subgroups (lymph nodes only vs. no lymph nodes)

➔ Details are listed below

Conclusion:

Absent influence of pathology on our results in the given setting.

Detailed results and graphs:

ADC_1

Sample 1	
Variable	ADC_1_LN
Sample 2	
Variable	ADC_1_wo_LN

	Sample 1	Sample 2
Sample size	32	33
Lowest value	0,3210	0,3120
Highest value	1,9940	2,5400
Median	1,0800	0,9980
95% CI for the median	0,9670 to 1,2841	0,9142 to 1,2138
Interquartile range	0,8500 to 1,3595	0,8733 to 1,3153

Average rank of first group	33,9688
Average rank of second group	32,0606
Mann-Whitney U	497,00
Test statistic Z (corrected for ties)	0,407
Two-tailed probability	**P = 0,6842**

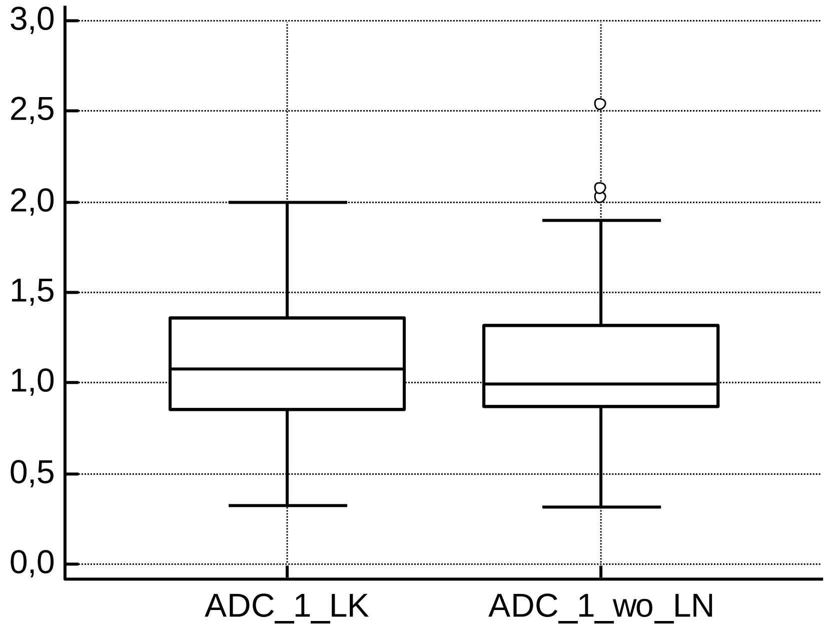

Figure 3: ADC_1 stratified by pathology (lymph node cases vs. non lymph node cases: LK vs wo_LN). ADC (y-axis) are given in [10^{-3} mm^2/s].

ADC_2

Sample 1	
Variable	ADC_2_LN
Sample 2	
Variable	ADC_2_wo_LN

	Sample 1	Sample 2
Sample size	32	33
Lowest value	0,4640	0,3130
Highest value	2,1330	2,7440
Median	1,1235	1,0560
95% CI for the median	0,9639 to 1,2371	0,9816 to 1,2815
Interquartile range	0,8955 to 1,4065	0,9050 to 1,4960

Average rank of first group	32,7812
Average rank of second group	33,2121
Mann-Whitney U	521,00
Test statistic Z (corrected for ties)	0,0919
Two-tailed probability	P = 0,9268

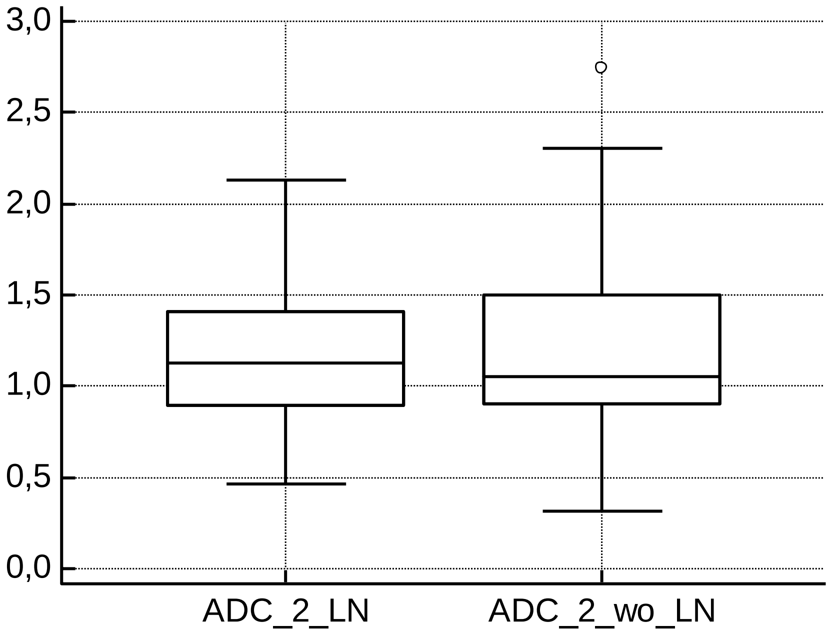

Figure 4: ADC_2 stratified by pathology (lymph node cases vs. non lymph node cases: LK vs wo_LN). ADC (y-axis) are given in [10^{-3} mm^2/s].

ADC_3

Sample 1	
Variable	ADC_3_LN
Sample 2	
Variable	ADC_3_wo_LN

	Sample 1	Sample 2
Sample size	32	33
Lowest value	0,4690	0,3150
Highest value	2,1290	2,7630
Median	1,1305	1,0550
95% CI for the median	0,9629 to 1,2531	0,9883 to 1,2756
Interquartile range	0,8935 to 1,4220	0,9038 to 1,4998

Average rank of first group	32,7344
Average rank of second group	33,2576
Mann-Whitney U	519,50
Test statistic Z (corrected for ties)	0,112
Two-tailed probability	P = 0,9112

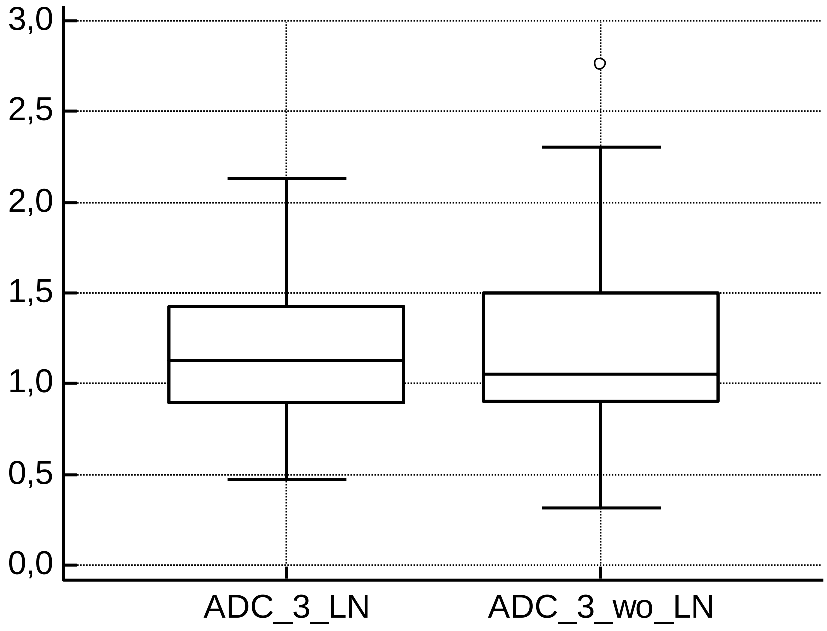

Figure 5: ADC_3 stratified by pathology (lymph node cases vs. non lymph node cases: LK vs wo_LN). ADC (y-axis) are given in [10^{-3} mm^2/s].

ADC_4

Sample 1	
Variable	ADC_4_LN
Sample 2	
Variable	ADC_4_wo_LN

	Sample 1	Sample 2
Sample size	32	33
Lowest value	0,4640	0,3170
Highest value	2,0360	2,5990
Median	1,0900	1,0250
95% CI for the median	0,9690 to 1,3011	0,9246 to 1,2348
Interquartile range	0,8600 to 1,3910	0,9055 to 1,3495

Average rank of first group	34,2344
Average rank of second group	31,8030
Mann-Whitney U	488,50
Test statistic Z (corrected for ties)	0,518
Two-tailed probability	P = 0,6042

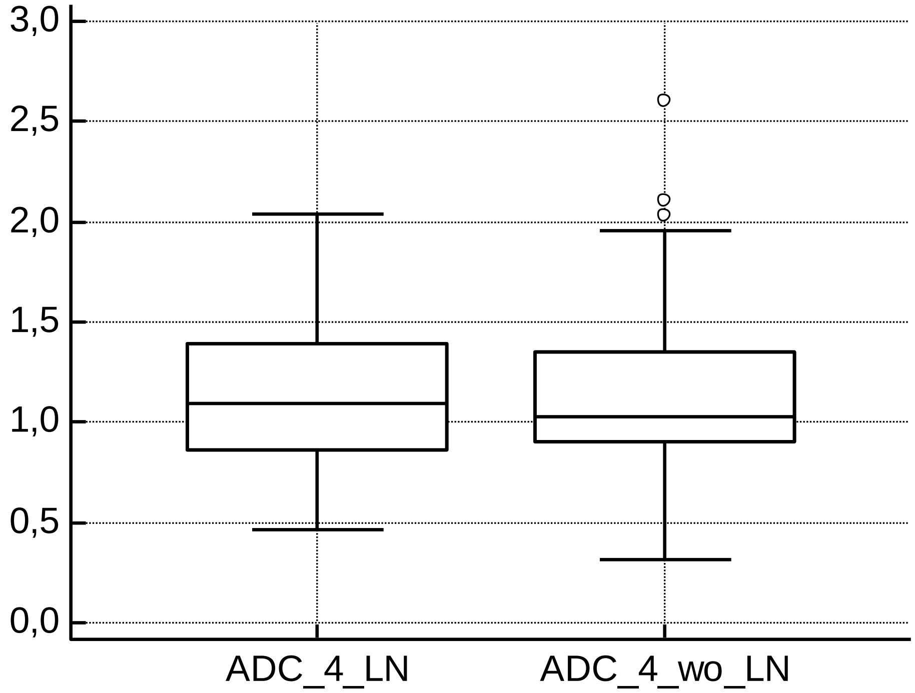

Figure 6: ADC_4 stratified by pathology (lymph node cases vs. non lymph node cases: LK vs wo_LN). ADC (y-axis) are given in [10^{-3} mm^2/s].

Part II

Lymph node cases only

	Minimum	25th percentile	Median	75th percentile	Maximum
ADC_1	0,3210	0,850	1,080	1,360	1,994
ADC_2	0,4640	0,896	1,123	1,406	2,133
ADC_3	0,4690	0,894	1,131	1,422	2,129
ADC_4	0,4640	0,860	1,090	1,391	2,036

Variable	Different (P<0,05) from variable nr
(1) ADC_1	(2) (3) (4)
(2) ADC_2	(1) (4)
(3) ADC_3	(1) (4)
(4) ADC_4	(1) (2) (3)

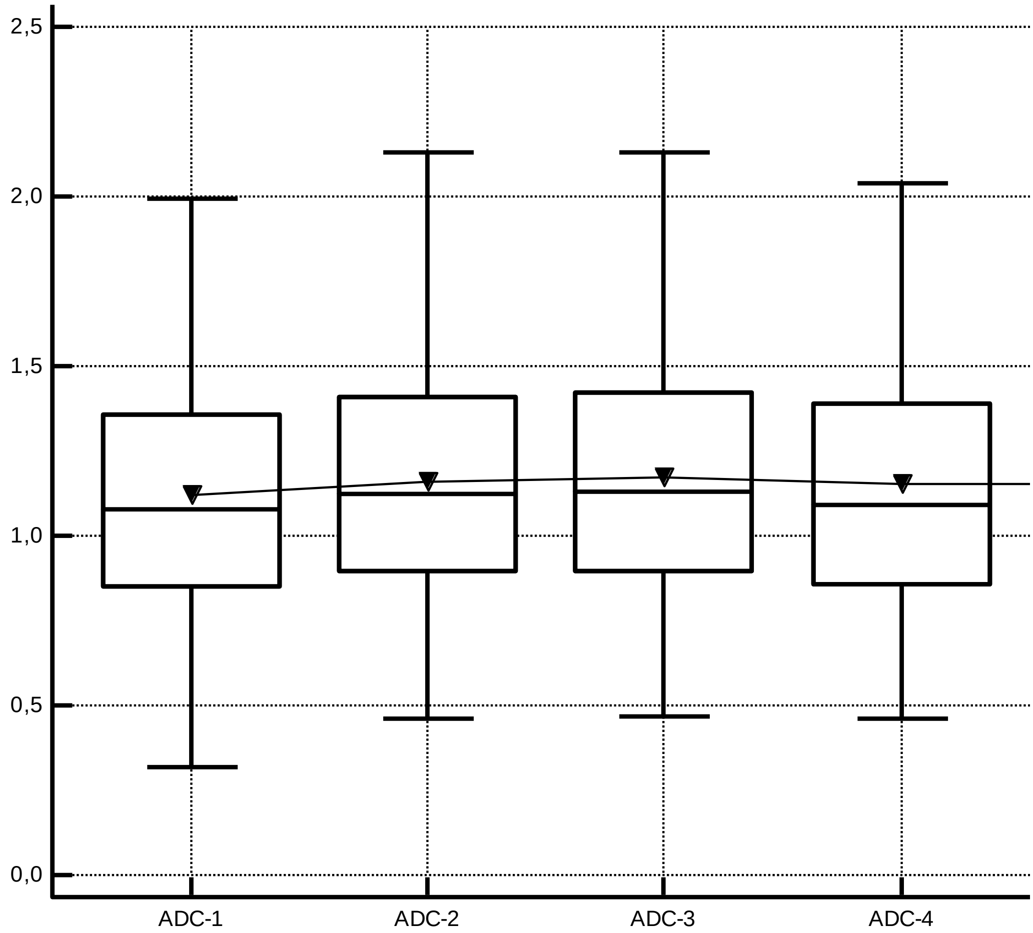

Figure 7: Lymph node cases stratified by PPM. ADC (y-axis) are given in [10^{-3} mm^2/s].

Non lymph node cases only

	Minimum	25th percentile	Median	75th percentile	Maximum
ADC_1	0,3120	0,873	0,998	1,315	2,540
ADC_2	0,3130	0,905	1,056	1,496	2,744
ADC_3	0,3150	0,904	1,055	1,500	2,763
ADC_4	0,3170	0,906	1,025	1,349	2,599

Variable	Different ($P<0,05$) from variable nr
(1) ADC_1	(2) (3) (4)
(2) ADC_2	(1) (4)
(3) ADC_3	(1) (4)
(4) ADC_4	(1) (2) (3)

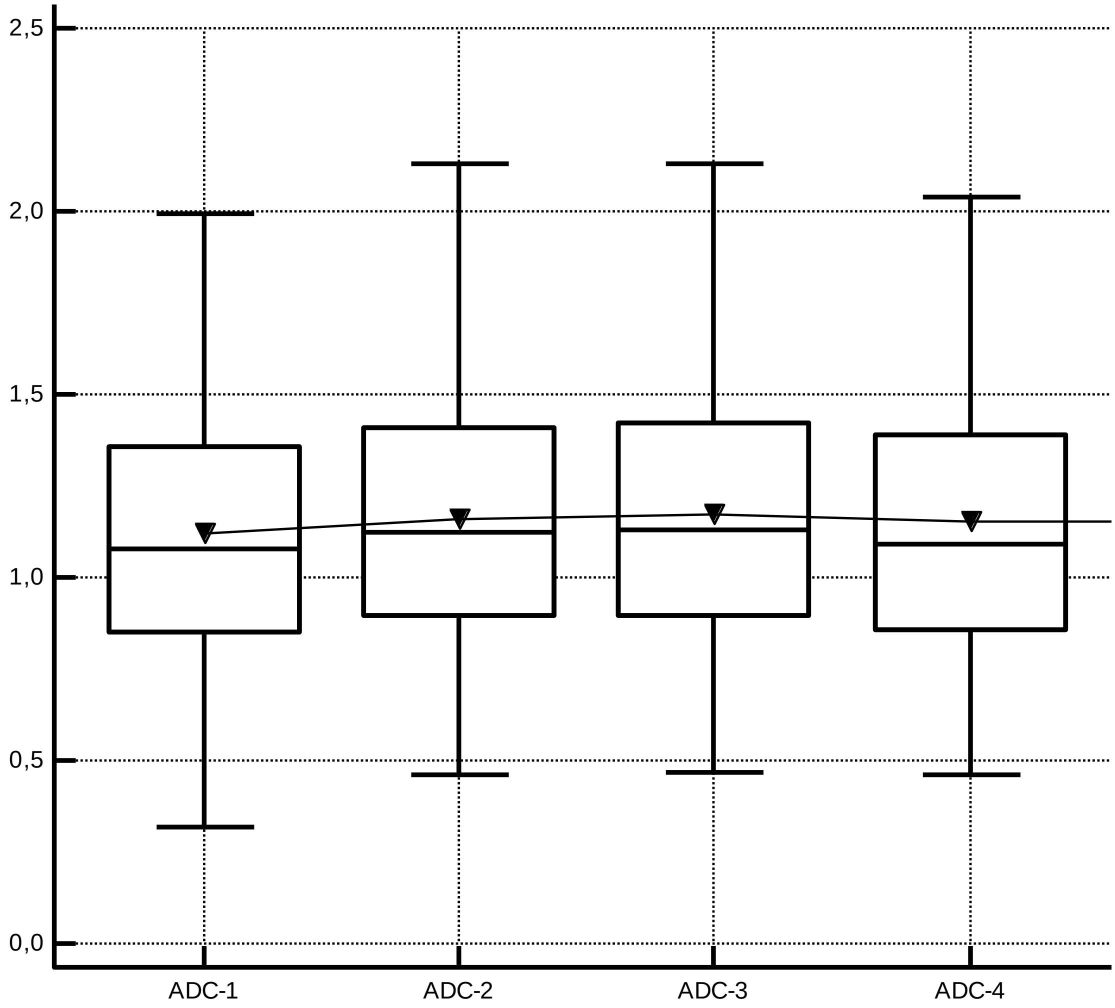

Figure 8: Non lymph node cases stratified by PPM. ADC (y-axis) are given in [10^{-3} mm^2/s].

References

[1] D. Le Bihan, É. Breton, Imagerie de diffusion in vivo par résonance magnétique nucléaire, C R Acad Sc Paris. (1985) 1109 – 1112.

[2] D. Le Bihan, E. Breton, D. Lallemand, P. Grenier, E. Cabanis, M. Laval-Jeantet, MR imaging of intravoxel incoherent motions: application to diffusion and perfusion in neurologic disorders, Radiology. 161 (1986) 401–407.

[3] R.M. El Kady, A.K. Choudhary, R. Tappouni, Accuracy of apparent diffusion coefficient value measurement on PACS workstation: A comparative analysis, AJR Am. J. Roentgenol. 196 (2011) W280–284.

[4] A.-M. Legendre, Nouvelles méthodes pour la détermination des orbites des comètes, Firmin Didot, 1805.

[5] C.F. Gauss, Theoria motus corporum coelestium in sectionibus conicis solem ambientium, Frid. Perthes et I. H. Besser, 1809.

[6] P.M. Schulze, D. Porath, Statistik: mit Datenanalyse und ökonometrischen Grundlagen, 7th ed., Oldenbourg, München, n.d.

[7] Y.M. Park, Y.Y. Byun, Understanding the Mathematics Involved in Calculating Apparent Diffusion Coefficient Maps, AJR Am J Roentgenol. 199 (2012).

[8] W. Conver, Practical nonparametric statistics, 3rd ed., Wiley & Sons, New York, 1999.
